# Vaccinating with conserved *Escherichia coli* antigens does not alter the mouse intestinal microbiome

**DOI:** 10.1186/s13104-016-2208-y

**Published:** 2016-08-11

**Authors:** Michael P. Hays, Aaron C. Ericsson, Yang Yang, Philip R. Hardwidge

**Affiliations:** 1 College of Veterinary Medicine, Kansas State University, Manhattan, KS 66506 USA; 2 University of Missouri (MU) Mutant Mouse Resource and Research Center, College of Veterinary Medicine, University of Missouri, Columbia, MO 65203 USA

## Abstract

**Background:**

Enterotoxigenic *Escherichia coli* (ETEC) causes diarrheal disease. Antigenic and structural heterogeneity among ETEC colonization factors has complicated vaccine development efforts. Identifying and characterizing conserved ETEC antigens that induce protective immunity is therefore of interest. We previously characterized three proteins (MipA, Skp, and ETEC_2479) that protected mice in an intranasal ETEC challenge model after vaccination. However, these proteins are conserved not only in multiple ETEC isolates, but also in commensal bacteria. While the impact of inactivated viral vaccines and live-attenuated bacterial vaccines on the host microbiota have been examined, the potential impact of using subunit vaccines consisting of antigens that are also encoded by commensal organisms has not been investigated.

**Findings:**

We addressed this issue by characterizing changes to mouse intestinal microbiomes as a function of vaccination. We failed to observe significant changes to mouse health, to mouse weight gain as a function of time, or to the diversity or richness of mouse intestinal microbiomes, as measured by analyzing alpha- and beta-diversity, as well as overall community structure, before and after vaccination.

**Conclusions:**

We conclude that despite the conservation of MipA, Skp, and ETEC_2479 among Gram-negative bacteria, vaccination with these antigens fails to alter significantly the host intestinal microbiome.

## Background

Enterotoxigenic *Escherichia coli* (ETEC) causes hundreds of millions of cases of diarrhea annually, particularly in developing countries [1]. In addition to their acute impact on human health, repeated infections also contribute to delayed growth and malnutrition [1]. Many vaccine strategies have focused on ETEC colonization factors (CFs). These heterogeneous surface structures function in attachment by binding to host glycoprotein conjugates [2]. Numerous CFs have been described, but many ETEC strains do not produce a recognizable CF [3]. A need therefore exists to identify new vaccine targets that are independent of strain-specific CFs.

We previously characterized three ETEC H10407 proteins as protective antigens in a mouse model involving intranasal bacterial challenge [4]. Antisera raised against the ETEC MipA, Skp, and ETEC_2479 proteins protected HCT-8 cells from attachment by multiple ETEC strains [4]. Immunization with these antigens also protected mice from an otherwise lethal challenge with intranasally administered ETEC H10407 [4]. Skp is a molecular chaperone that rescues misdirected outer membrane proteins [5]. MipA is an immunoreactive protein [6] that belongs to a family of proteins involved in remodeling peptidoglycan. ETEC_2479 is predicted to function as an outer membrane porin involved in long chain fatty acid transport [7]. The intranasal challenge model [8, 9] is a useful alternative for ETEC vaccine studies because mice do not naturally develop diarrheal disease after oral ETEC challenge [10]. This model also permits the quantification of mouse survival, bacterial clearance, and host immune responses, and stimulates mucosal immune responses, especially secretory IgA (sIgA) responses that are important to blocking bacterial adherence to mucosal surfaces.

Identifying broadly conserved, protective antigens is important to vaccine development. However, MipA, Skp, and ETEC_2479 are conserved not only among pathogenic ETEC strains, but also among the commensal *Proteobacteria*. This may be an important issue because it is known that alteration of commensals can influence susceptibility to gastrointestinal disease [11] and vaccine efficacy [12]. Several previous studies have begun to address this issue. Rotavirus vaccination of humans did not have a major impact to infant microbiomes [13]. A challenge of cynomolgus macaques with an oral-live attenuated *Shigella* strain found a possible protective role for the microbiota and highlighted the importance of considering host genetics in vaccine studies [14]. Oral immunization with the live-attenuated typhoid vaccine strain Ty21a did not cause significant perturbation of the fecal microbiota related to vaccine administration [15]. Thus, while the impact of inactivated viral vaccines and live-attenuated bacterial vaccines on the host microbiota have been examined, the potential impact of using subunit vaccines consisting of antigens that are also encoded by commensal organisms has not been investigated.

We hypothesized that using the conserved antigens MipA, Skp, and ETEC_2479 as subunit vaccine candidates could negatively impact the health of the host by affecting the intestinal microbiota. Herein, we tested and subsequently refuted this hypothesis.

## Methods

### Ethics statement

Animal experiments were performed according to Kansas State University Institutional Animal Care and Use Committee-approved protocols (IACUC #3648). This institution complies with all applicable provisions of the Animal Welfare Act and other Federal statutes and regulations relating to animals.

### Antigen purification

*Escherichia coli* BL21(DE3) strains expressing individual antigens were grown overnight as described [4]. After purification, proteins were dialyzed into glycerol in Pierce Slide-A-Lyzer dialysis cassettes. Protein concentrations were quantified by using the Precision Red Advanced Protein Assay (Cytoskeleton, Inc.).

### Polyclonal antisera production

Female BALB/c mice of matched age (6 weeks at initial vaccination) were obtained from the Jackson Laboratory (Bar Harbor, Maine) and handled as described previously [4]. Mice were housed (5 per group) in microisolator cages (1 cage per group) and provided with food and water ad libitum. Antigens (20 µg/dose) were mixed with 2.5 µg of cholera toxin in 25 µl phosphate-buffered saline (PBS) and then administered intranasally to the external nares of mice that had been anesthetized with isoflurane [4]. Two booster doses were administered, at 2- and 4-weeks after the initial vaccination. Mice were euthanized 2 weeks after the final immunization and exsanguinated. The blood was processed into serum using centrifugation for 2 min at 2500*g* in a BD microcontainer serum separator tube. Control serum samples were also obtained from mice immunized with PBS or with EHEC EspB.

### Immunoassays

Enzyme-linked immunosorbent assays (ELISAs) were performed as previously described [4], using serial dilutions of mouse serum samples and polystyrene 96-well, flat bottom plates (Whatman) coated with purified antigens or with bovine serum albumin (BSA; 0.5 µg/ml). Plates were developed with 1-StepTM Ultra TMB-ELISA (Thermo) and quenched with 3 N H_2_SO_4_. Absorbance was read at 450 nm.

### Fecal DNA extraction

A fecal pellet from each mouse was collected weekly, with the initial collection prior to the first vaccination and the final collection 2 weeks after the final vaccination. After collection, the fecal pellets were stored at −80 °C until DNA extraction could be performed. Prior to DNA extraction using QIAamp DNA Stool Mini Kits (Qiagen), 1.4 ml of Buffer ASL was added to fecal samples on ice. Fecal pellets were vortexed until completely resuspended and DNA was extracted by following the manufacturer’s protocol.

### Library construction and sequencing

Library construction and sequencing was performed essentially as described [16]. Prior to PCR, DNA concentrations were determined via fluorometry (Qubit dsDNA BR assay, Life Technologies, Carlsbad, CA) and normalized to a standard concentration. Bacterial Microbial 16S rRNA amplicons were generated via amplification of the V4 hypervariable region of the 16S rRNA gene using single-indexed universal primers as described previously [U515F/806R; 17] flanked by Illumina standard adapter sequences. Primer sequences are available at proBase [18; http://www.microbial-ecology.net/probebase/]. PCR was performed using the following parameters: 98 °C^(3:00)^ + [98 °C^(0:15)^ + 50 °C^(0:30)^ + 72 °C^(0:30)^] × 25 cycles +72 °C^(7:00)^. Following PCR, amplicons were pooled for sequencing using the Illumina MiSeq platform and V2 chemistry with 2 × 250 bp paired-end reads.

### Informatics analysis

Informatics analysis was performed essentially as described [16]. FLASH software was used to assemble contiguous DNA sequences [18]. Sequences were culled if determined to be short after trimming for a base quality less than 31. Reference-based and *de novo* chimera detection and removal was conducted using Qiime v1.8 software [19]. Remaining contiguous sequences were assigned to operational taxonomic units (OTUs) via *de novo* clustering with a criterion of 97 % nucleotide identity as described [16]. Annotation of selected OTUs was performed using BLAST [20] against the Greengenes database [21] of 16S rRNA sequences and taxonomy. Principal component analysis was performed using ¼ root-transformed OTU relative abundance data via a non-linear iterative partial least squares (NIPALS) algorithm, implemented using an open access Excel macro available from the Riken Institute (http://prime.psc.riken.jp/Metabolomics_Software/StatisticalAnalysisOnMicrosoftExcel/index.html). Sequence data were deposited in the NCBI Sequence Read Archive (SRA) under the BioProjectID PRJNA320839.

### Statistical analyses

Statistical analysis was performed using Sigma Plot 12.3 (Systat Software Inc., Carlsbad, CA). Interactions and differences between treatment groups and time-points in Chao1 indices were determined using 2-way analysis of variance (ANOVA). Analysis of molecular variance (AMOVA) was implemented in a general linear model using SPSS software, version 23 (IBM, Armonk, NY). Results were considered statistically significant for *p* values ≤0.05.

## Results

**Fig. 1:**
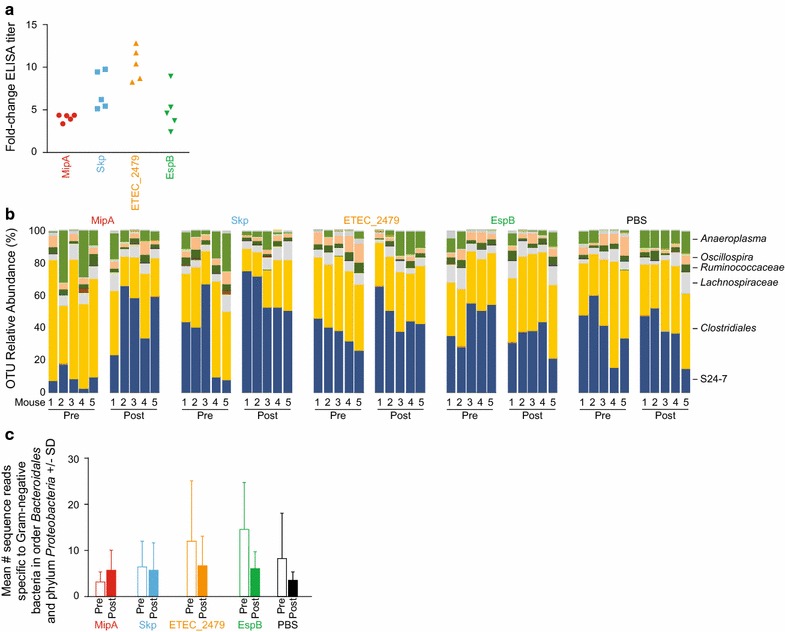
Vaccination with ETEC MipA, Skp, and ETEC_2479. **a** Serum IgG responses in mice. Data are plotted as the fold-change in serum IgG after immunization with the indicated antigens, n = 5/group. **b** Bar chart showing relative abundance of all operational taxonomic units (OTUs) detected in the feces of mice prior to (pre) and 6 weeks after (post) vaccination with the indicated antigens, as detected using 16S rRNA amplicon sequencing. The identities of dominant taxa are shown at the right. **c** Mean number + standard deviation (SD) of sequence reads that were specific to *Bacteriodales* or *Proteobacteria* in indicated treatment groups

Mice were vaccinated intranasally with purified, recombinant forms of ETEC MipA, Skp, and ETEC_2479 [4], as well as with a purified, recombinant form of *E. coli* O157:H7 EDL933 EspB [22]. EspB was used as an external control because it is immunogenic but is not expressed by either ETEC or by commensal bacteria [23]. After 3 immunizations, mice were sacrificed and their serum was used in ELISAs to quantify antibody titers. All mice (5/group) produced detectable IgG titers (Fig. 1a). Mouse health and weight gain were monitored during the vaccination regimen. Neither obvious changes to mouse health or behavior, nor changes in the rate of weight gain were observed. The weights of the mice after vaccination were 21.1 ± 1.0 g for the PBS control group, 20.7 ± 1.0 g for EspB, 21.2 ± 0.9 g for MipA, 21.2 ± 2.0 g for Skp, and 21.3 ± 0.9 g for ETEC_2479 (p > 0.05). Fecal IgA responses for mice immunized with either MipA, Skp, or ETEC_2479 ranged from a 13.2 ± 2.2, 13.0 ± 3.4, and 26.8 ± 4.6 fold-increase as compared with control groups, respectively [4].

**Fig. 2:**
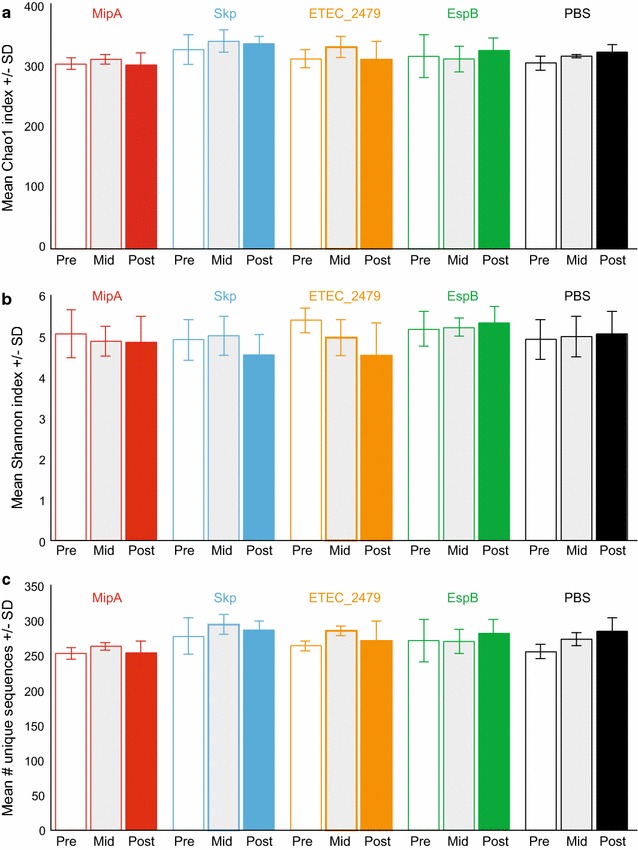
Sequence diversity among treatment groups. **a** Bar chart showing mean + standard deviation (SD) Chao1 a-diversity index of fecal microbiota in mice pre- and post-vaccination with the indicated antigens, as detected using 16S rRNA amplicon sequencing. **b** Mean Shannon diversity indices. **c** Mean number of unique sequences in treatment groups

To determine whether vaccination affected the diversity and overall composition of the mouse intestinal microbiota, 16S rRNA amplicon sequencing was performed using DNA extracted from feces collected prior to vaccination, from the mid-point of the vaccination regimen (after the second vaccination), and two weeks after the final vaccination, as template. Following vaccination, there were no apparent differences in the microbial profiles when resolved to the level of operational taxonomic unit (OTU), and the same six OTUs (families S24-7, *Lachnospiraceae*, *Ruminococcaceae*, order *Clostridiales*, *Oscillospira* sp., and *Anaeroplasma* sp.) dominated the pre- and post-vaccination microbiota of all groups (Fig. 1b). Regarding the effect of vaccination on other Gram-negative taxa that potentially express the targeted antigens, i.e., microbes in the order *Bacteroidales* or phylum *Proteobacteria*, no differences were detected between pre- and post-vaccination samples in the relative abundance of these bacteria (Fig. 1c). The Chao1 index, a measure of α-diversity (i.e., within samples), was also compared between groups to determine if vaccination affected the richness or distribution of microbes. Two-way ANOVA detected no significant differences between groups, or between pre- and post-vaccination samples (Fig. 2a). Samples obtained from the mid-point of the vaccination regimen (after the second vaccination) were also analyzed and were not significantly different from pre-or post-vaccination samples (Fig. 2a). Similarly, neither the Shannon diversity index, nor the raw number of unique sequences detected in each group, were significantly different among groups (Fig. 2b, c).

**Fig. 3:**
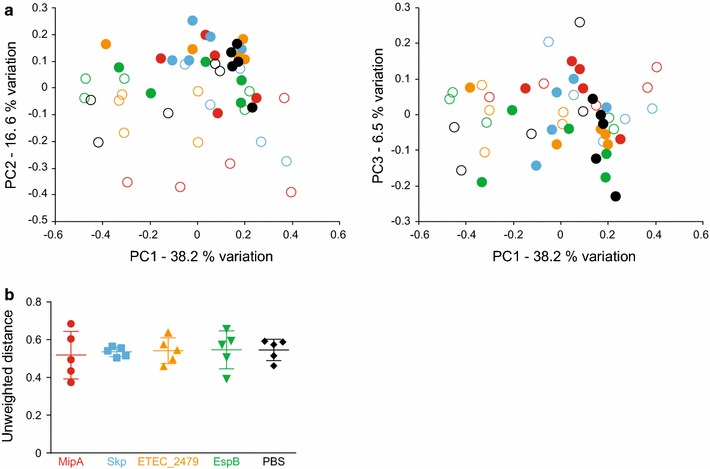
Principal component analyses and UniFrac distances. **a** Unweighted principal component analyses showing β-diversity of fecal microbiota in mice pre- and post-vaccination with the indicated antigens, as detected via 16S rRNA amplicon sequencing. Principal component 1 (PC1) versus PC2 (*left*) and PC1 versus PC3 (*right*) are shown. *Color-coding* is identical to Fig. 2, with *open symbols* representing pre-vaccination and *closed symbols* representing post-vaccination samples. **b** Mean intragroup unweighted UniFrac distances between pre- and post-vaccination samples

Principal component analysis (PCA) was performed both to assess β-diversity (i.e., between samples) and to determine if vaccination induced changes in the overall composition of the microbiota. No clustering of post-vaccination samples was observed in plots of principal component 1 (PC1) against PC2 or PC3, which accounted collectively for over 60 % of the variability between samples, suggesting that there were negligible shifts in the bacterial community composition of any treatment group (Fig. 3a). A comparison of the mean intragroup unweighted UniFrac distance between pre- and post-vaccination samples detected no greater dissimilarity between the two samples in treated groups as compared to the control group, as determined by performing ANOVA with post hoc Dunnett’s tests (Fig. 3b). Supporting our prior analysis, AMOVA detected no significant effect of vaccination treatment (p = 0.053; F value = 3.302), and also no significant effect of time-point (p = 0.359; F value = 4.517), using the first and last samples as time-points.

## Discussion

The discovery and characterization of broadly conserved ETEC vaccine antigens that are independent of strain-specific CFs is of emerging interest. However, an important consideration is whether targeting antigens also expressed by commensal flora will negatively impact host health or vaccine efficacy. We have characterized the ETEC proteins MipA, Skp, and ETEC_2479 for their protective efficacy in an intranasal challenge model. These proteins are highly conserved not only in diverse ETEC isolates, but also in commensal *Proteobacteria* and other *E. coli* and *Shigella* strains, sharing ~99 % identity with the corresponding *Shigella* proteins. Because altering commensal abundance and diversity may affect host health and vaccine efficacy, we were therefore interested in determining whether using these antigens in a subunit vaccine would affect the mouse microbiota. Commensal *E. coli* strains may contribute to colonization resistance against pathogens [24]. For example, *E. coli* Nissle 1917 has been extensively characterized as a probiotic agent [25] and could potentially function by competing for nutrients that are required by pathogens [24]. They also play important, though incompletely defined roles in maintaining intestinal homeostasis [26].

We did not observe changes to mouse health, behavior, or rate of weight gain following intranasal vaccination with either MipA, Skp, or ETEC_2479. We also observed no significant differences among the microbial profiles when resolved to the level of operational taxonomic unit (OTU), as determined by performing 16S rRNA amplicon sequencing. Analysis of the Chao1 index, the Shannon diversity index, and the raw number of unique sequences detected in each treatment group, also failed to reveal any significant differences. PCA analysis and comparison of UniFrac distances also suggested negligible shifts in the bacterial community composition of any treatment group.

While no consistent shifts in the composition of the fecal microbiota were detected following vaccination against the three conserved candidate antigens, a closer examination of the effects of vaccination on the relative abundance of taxa expected to express these antigens, i.e., bacteria within order *Bacteroidales* and phylum *Proteobacteria*, was performed. While no vaccination-dependent differences were detected in the relative abundance of these groups, we recognize that both *Bacteroidales* and *Proteobacteria* are rare taxa in the current samples. This is not however unique to this cohort of mice, as we have demonstrated very comparable levels of these taxa in multiple genetic backgrounds of mice purchased from the same vendor and the overwhelming majority of gut bacteria in most research mice are Gram-positive [27]. Regardless, the low numbers of Gram-negative bacteria present in the present cohorts were not affected by vaccination. It will be important in future studies to validate these studies using diarrheal disease models for ETEC in either mice or piglets.

Overall, despite the conservation of MipA, Skp, and ETEC_2479 among Gram-negative bacteria, vaccination with these antigens fails to alter significantly the host intestinal microbiota and suggests that their inclusion in future ETEC vaccine preparations may be efficacious.

## Abbreviations

AMOVA: analysis of molecular variance
ANOVA: analysis of variance
BSA: bovine serum albumin
ELISA: enzyme-linked immunosorbent assay
ETEC: enterotoxigenic *Escherichia coli*
OTU: operational taxonomic unit
PBS: phosphate-buffered saline
PCA: principal component analysis
sIgA: secretory IgA
